# Supplementary material for: How to evaluate a multi-country implementation-focused network: Reflections from the Quality of Care Network (QCN) evaluation
Source: PLOS Glob Public Health. 2024 Aug 29;4(8):e0001897. doi: 10.1371/journal.pgph.0001897 (PMC11361611; doi:10.1371/journal.pgph.0001897)
Supplement: Attachment — Submitted filename: Response_to_ Reviewers.pdf [file pgph.0001897.s007.pdf]

PGPH-D-23-00381R1

How to evaluate a multi-country implementation-focused network: Lessons from the Quality of Care Network (QCN) evaluation

Dear Editor and Reviewers,

Thank you for your comments and the opportunity to further revise our paper. Please see our responses below each comment raised and the uploaded tracked changes version of our paper.

We look forward to hearing from you soon, and thank you in advance.

Yours sincerely,

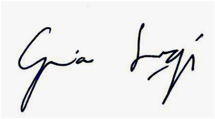

Gloria Seruwagi, on behalf of all authors.

Please consider revising the title of the paper. It suggests that the paper is methodological in nature, advising how to evaluate a network, whereas the content is reflecting on the network.

Thank you for this comment. On receiving this last set of review comments, we immediately followed up with an enquiry – via email (dated 11 January 2024) – which, partly, was in response to this comment. As a team we felt that we need to hear back from the editor before we agree on a new title given it looks like it had not been fully understood this time round – and it was the first time in more than five rounds of paper revisions that the issue of topic clarity was being brought up. We think that the original title is clear that the paper is about how we evaluated the network (and lessons learnt in terms of evaluation methods and the way we went about out study) not lessons from the network itself, but from our work evaluating it. We could change it to another title if absolutely necessary – after hearing back from you – from "*How to evaluate a multi-country implementation-focused network: Lessons from the Quality of Care Network (QCN) evaluation*" to "*Key Lessons from evaluating the World Health Organisation's multi-country Quality of Care Network (QCN)*" and await your final guidance on this matter.

Please also revise the abstract. Couch the background section of the abstract into the warrant of the paper - why is this important, in relation to other studies?

This comment has been addressed, and the importance of this paper in relation to other studies highlighted (page 2, lines 32-33)

Please clarify the methods section again as suggested by reviewers.

The two review comments received on the methods section are:

- Reviewer #1: The method section remains a bit unclear and would benefit from more structure, for example like the descriptions in S2. For example, how where the questions asked, by whom and to whom, online, in person, questionnaire, focus group? What analysis did you use? As written now, the method section is not

reproducible. The result section is a lot of text and would benefit from some table and/or figures to clarify which stakeholders where involved and what the process looked like etc. Please check some of the phrases for grammatical mistakes.

- Reviewer #2: The paper is a lot more cohesive now and it is easy to see the rationale, method, results, and discussion. You've also addressed the continuity issues I raised.

In response:

- We very much appreciate Reviewer #1 comments and have provided more structure, descriptions and visuals as advised (pages 7 -16. Visuals can be found on page 7 line 179, page 8 line 203 and page 9 lines 215 – 216)
- The previous version had sections explaining and answering some of Reviewer #1's comments – specifically on questions asked and analysis (see for example page 7, lines 162-167; 172 – 174). However, we have added other details and believe it is now reproduceable as written. We have visualized some of the findings as advised – for example by a table showing the main themes that came out (Page 9, line 215 - 217) and have also now included a figure encapsulating our evaluation team (page 8 line 203 and the figure is separately attached in line with journal guidelines
- We have undertaken additional checks and editing to ensure that there are no grammatical mistakes.
- Good to see that Reviewer#2 is happy with the paper.

Please add relevant international network literature into the paper, so the reader understands more how this paper furthers the field in understanding such collaborations.

Kindly please note that we have already discussed our paper in relation to similar papers on "how to evaluate a network" (pages 18-19, lines 443-457, references 11-18 already listed in the paper). We have added one sentence at the end of this paragraph (page 19 line 457-458) to clarify that these lessons are relevant to both evaluations, and to network collaborations themselves.

Please also note that our paper is part of a collection and our main papers reporting the results of our evaluation cover relevant literature on international networks themselves, including this already published paper by Akter et al:
Akter K, Shawar Y, Tesfa A, et al. Influences on policy-formulation, decision-making, organisation and management for maternal, newborn and child health in Bangladesh, Ethiopia, Malawi and Uganda: the roles and legitimacy of a multi-country network *PLOS Global Public Health* 2023; 3(11): e0001742

- Our paper by Djellouli et al, which is in press with PLOS Global Public Health, and pre-printed here:
Djellouli N, Shawar YR, Mwaba K, et al. Effectiveness of a multi-country implementation-focused network on quality of care: delivery of interventions and processes for improved maternal, newborn and child health outcomes *PLOS Global Public Health 2024* (in press);
https://www.medrxiv.org/content/10.1101/2023.03.03.23286747v1

- and our paper by Shawar et al, which is still under review, and pre-printed here:
Shawar Y, Djellouli N, Shiffman J, et al. Factors Shaping Network Emergence: A Cross-Country Comparison of Quality of Care Networks in Bangladesh, Ethiopia, Malawi & Uganda *PLOS Global Public Health* 2024 (under review).
https://www.medrxiv.org/content/10.1101/2023.03.29.23287925v1
