# Supplementary material for: How to evaluate a multi-country implementation-focused network: Reflections from the Quality of Care Network (QCN) evaluation
Source: PLOS Glob Public Health. 2024 Aug 29;4(8):e0001897. doi: 10.1371/journal.pgph.0001897 (PMC11361611; doi:10.1371/journal.pgph.0001897)
Supplement: Attachment — Submitted filename: 1aa_Cover_Letter_Response to Reviewers_ 20072023.docx [file pgph.0001897.s005.docx]

**Response to Reviewers: Manuscript Reference PGPH-D-23-00381**

The Editorial Team

PLOS Global Public Health

Dear Editor,

We were so grateful to receive feedback on our manuscript titled “*How to evaluate a multi-country implementation-focused network: Lessons from the Quality of Care Network (QCN) evaluation”.* We thank the reviewer for the time taken and valuable critique to improve our manuscript.

Below we provide a point-by-point response to the comments raised:

**Journal Requirements:**

1. Please provide separate figure files in .tif or .eps format only and remove any figures embedded in your manuscript file. Please also ensure all files are under our size limit of 10MB. This has been done.

2. We have noticed that you have uploaded Supporting Information files, but you have not included a list of legends. Please add a full list of legends for your Supporting Information files after the references list. This has been done (Line 791 -798)

3. In the online submission form, you indicated that "Relevant data has been submitted alongside this manuscript and additional data is available on request". All PLOS journals now require all data underlying the findings described in their manuscript to be freely available to other researchers, either 1. In a public repository, 2. Within the manuscript itself, or 3. Uploaded as supplementary information. This policy applies to all data except where public deposition would breach compliance with the protocol approved by your research ethics board. If your data cannot be made publicly available for ethical or legal reasons (e.g., public availability would compromise patient privacy), please explain your reasons by return email and your exemption request will be escalated to the editor for approval. Your exemption request will be handled independently and will not hold up the peer review process, but will need to be resolved should your manuscript be accepted for publication. One of the Editorial team will then be in touch if there are any issues. We have now added critical data in the key areas – example in the methods section – and other information submitted separately is only supplementary, or as per journal requirements.

**Introduction**

- Line 71, is not clear what the aim was, too vague wording. This has been reworded (Line 71-72)
- Line 82, references or definition of pathfinder countries is missing. Pathfinder definition has been added the very time the word appears (Line 68-71)
- Line 88, this whole paragraph is confusing, what is this paper about? I would shorten this or mention the other papers in a box. This section has been removed and signposted to as it also already formed part of the supplementary information submitted with the manuscript.
- Line 133, what do you mean by Quality of Care (QoC) definition? QoC has been defined as per WHO guidelines (Line 105 - 107)
- Parts of what is described in "the QCN Evaluation" (line 151) belong in the method section. This has been addressed – the content moved to respective sections - and additional explanation given in the sections below.

**Methods**

- Are the methods about the content of this paper? Or of the whole project and all the papers? This has to be made clear.

**Response:** Both. Our methods section is divided into three sections: methods of the wider QCN evaluation (this is for the whole project and all of the papers), composition, characteristics and organization of the evaluation team, and methods for the reflections that underlie this report (the methods about the content of this paper).

- I am also missing some references to different methods used, the reader cannot be required to go to a different paper/supplement to understand the basic methods.

**Response:** Thank you for this comment, and apologies the methods were not clearly presented. Because this is a collection of nine papers from a single project, overlaps were bound to occur in the methods sections for most or all of the papers. It was agreed that we write an overarching Methods Supplement providing sufficient detail on evaluation methodology – which all the papers would signpost to. We have added some details (Line 247-250) to give the reader an idea of the scope of the methods used in the overall QCN evaluation.

- It also seems sometimes results/discussion items are mentioned in the methods, such as line 213 "the team was able to effectively implement the evaluation". And the final part of the methods section, starting at line 234 "The process we describe has limitations"

**Response:** As mentioned in the manuscript’s abstract and introductory sections, we approached this paper as a reflective piece, a learning paper and - as such - the structuring was not as clear cut for methods or results. Line 213 was trying to indicate some of the barriers overcome despite methodological process challenges encountered in the conduct of this evaluation, for example COVID-19. Same as line 234 which is a reflection on the limitations of our approach.

**Results**

- It is also not clear how the method section leads to the results described. Needs more linkage and the result section needs more context, why are these results discussed?

**Response:** The methods section predominantly focuses on process issues, and then builds on that to the “results” section which outlines the pros and cons of evaluation approach – opportunities, innovations, learnings, etc. As mentioned earlier, being a reflective piece, this paper might not neatly fit into the standard format of most manuscripts with a methods/results/discussion section; however, we have attempted to provide a logical flow which we hope is clearer after this explanation.
 **Discussion**

- The discussion should start by going back to original aim of the study. This relates to an earlier comment, it is not clear what this paper is describing. Make sure no "new" results are mentioned in the discussion.

**Response:** Very useful guidance – thank you very much. We have edited this section, provided clarity right at the beginning of the discussion section (Line 547 - 552).

We hope that that the review comments have been addressed and look forward to your positive consideration of our manuscript.

Yours sincerely,

*Gloria Seruwagi*

Makerere University – School of Public Health

Corresponding author, on behalf of the authors
