# Supplementary material for: How to evaluate a multi-country implementation-focused network: Reflections from the Quality of Care Network (QCN) evaluation
Source: PLOS Glob Public Health. 2024 Aug 29;4(8):e0001897. doi: 10.1371/journal.pgph.0001897 (PMC11361611; doi:10.1371/journal.pgph.0001897)
Supplement: Attachment — Submitted filename: QCN Paper 8 response to reviewers comments.docx [file pgph.0001897.s006.docx]

PGPH-D-23-00381R1

**How to evaluate a multi-country implementation-focused network: Lessons from the Quality of Care Network (QCN) evaluation**

Dear Editor and Reviewers,

Thanks for your comments and the opportunity to further revise our paper. Please see our responses below each comment and uploaded tracked changes version of our paper. We hope our revisions sufficiently satisfy the criteria for this manuscript to be considered a research article.

We look forward to hearing from you soon,

Gloria Seruwagi, on behalf of all authors.

**Reviewer #1:** As this is submitted as a Research Article I expect a clear distinction between the method and the result section but also a clear relation between them. It becomes a bit clearer why the results were chosen, but this should have also been mentioned at the beginning of the results section. As the authors indicate themselves the distinction between method and result is not clearly stated as it is more a reflective piece. I do feel that the authors should have another critical look and make sure that the methods only describe methods and no results.

**Authors response:** Thank you for your careful review of our paper. We have clarified and expanded our description of the methods we used to investigate our research process in this piece (we have added the questions we asked, and the methods we used to analyse the responses). We have also moved this expanded section up in our methods section to make it more prominent, and have taken the “Methods of the wider QCN Evaluation” section out to more clearly show that this paper is about investigating and reporting on our research process and learnings. We have also checked through our methods section and moved the “Composition, characteristics and organisation of the evaluation team” section to the Results, and have added two sentences at the end of our methods section to explain how our Results section is now structured. We have deleted the results that are not focused on our research process, and explained how the results are structured at the end of the methods section. We have also revised our abstract to reflect our revised methods section, and added a clearer sentence to the end of our introduction explaining what this paper is about.

**Reviewer #2:** This paper is a reflective piece about a 3.5-year multi-country, multi-disciplinary project. This paper is not really about quality of care for maternal health - the paper has almost nothing to say about that - so much as the operational challenges and learnings of organizing a multi-country project. I think there's some worthwhile points in the paper to this effect, but I think it should be presented differently.

As you say in your response to the previous reviewer, this paper doesn't fit well with the standard methods/results/discussion format of scientific reporting. I also agree that it doesn't work well in this format. So why have you chosen to present it this way? I thought the previous reviewer made some good points about the limitations of your manuscript along these lines, but you haven't addressed these points so much as dismissed them as being a consequence of the format you've chosen to adopt.

**Authors response:** Thank you for your review. We are indeed constrained by the standard research article format of the journal, though as per our response to Reviewer 1 above, have tried harder to make it fit this format as there are no other suitable article types available in the journal and we would like to keep this piece in the journal as part of our 9-paper QCN Evaluation collection. Please see above and below for the changes we have now made to do this.

The methods section is still confusing, as it briefly summarizes the methods for the project as a whole, and then the methods for this paper. The latter isn't very clear. It says the first author asked other team members to reflect on "primary questions". But you don't explain how you conducted this, nor clearly indicate what the primary questions are. By labelling this paper's approach as a method, you invite methodological scrutiny. The limitations to the methods should come later on in a limitations section.

**Authors response:** We have now expanded and clarified the methods for this paper, including adding the primary questions we asked, and the methods of analysis we used to analyse the responses to them. We have also removed the section of the methods for the project as a whole to more clearly show the focus of this paper on investigating our research process. We hope this section now clearly indicates what the primary questions are and how we conducted this study. We have moved the limitations of these methods from the methods section to our discussion section.

There's also some lack of continuity in this paper. For example, co-production is discussed over two paragraphs in the discussion, but is not in the methods or results sections. The balance between standardization and contextual adaption is mentioned as a key finding in the abstract and conclusion, but this finding isn't sufficiently developed in the results or discussion sections.

**Authors response:** Thank you for bringing these issues to our attention, we have brought in the issue of co-production both to our methods and our results sections. We have also added detail on the balance between standardisation and contextual adaptation to the results section and added a discussion of these results. It is presented between lines 378-401.

Ultimately, I feel that you haven't taken the previous reviewer's comments seriously enough, which is the main reason for my decision as they raised similar concerns to mine. I think a good way forward could potentially be to rewrite this as more of a discussion/commentary piece (i.e. not following the methods/results/discussion format) and reconsider where you submit it.

**Authors response:** Please see our responses above and significant revisions to the paper. We hope the paper now fits the research article format as we would like to keep this piece in the journal as part of our 9-paper QCN Evaluation collection and the PLOS GPH journal does not offer any other suitable article types.
