# Supplementary material for: How to evaluate a multi-country implementation-focused network: Reflections from the Quality of Care Network (QCN) evaluation
Source: PLOS Glob Public Health. 2024 Aug 29;4(8):e0001897. doi: 10.1371/journal.pgph.0001897 (PMC11361611; doi:10.1371/journal.pgph.0001897)
Supplement: Attachment — Submitted filename: Response to Reviewer_31052024.docx [file pgph.0001897.s008.docx]

**Manuscript: PGPH-D-23-00381R3**

**How to evaluate a multi-country implementation-focused network: Lessons from the Quality of Care Network (QCN) evaluation**

**Response to Review Comments**

1. Clarify the Research Question: The fundamental research question is not clear. On one hand, the manuscript suggests that it is attempting to demonstrate if this evaluation method is better, worse, or equally effective as other evaluation methods. On the other hand, this manuscript is attempting to explain the value of this specific network.

**Response:** We thank the reviewer for this comment. For clarification, this paper is not attempting to compare our evaluation approach with others (if it was better, worse or equally effective); rather it is a reflective methodological piece – as we state at the end of our introduction section: “In this paper we investigate, report on, and reflect on our research process, and learning gained from its conduct.”

2. Compare this Evaluation to Others: The authors provide detail about what they did, yet there is little comparison of this evaluation method contrasted against others. Incorporating and discussing relevant international network literature could significantly enrich the paper. It would place the QCN evaluation within a broader context, helping to underscore how this paper advances the understanding of evaluating such networks and collaborations.

**Response:** Grateful for this comment, too. In our discussion we do cite other literature that uses related methods and approaches to evaluating networks and related implementation-focused efforts – see paragraphs three to five of our discussion, and references 10-34.
As explained above, it was not our intention to compare this evaluation to others – although we aimed to contribute to the currently pauce literature on evaluating such complex networks using a multi-disciplinary approach. This manuscript is part of a collection of nine (9) other papers in this journal - most already published - and we do have a specific paper, by Shawar et al (close to acceptance at PLOS Global Public Health, but available as a preprint here: <https://www.medrxiv.org/content/10.1101/2023.03.29.23287925v1.full.pdf> ) which delves deeper into international network literature, including related theoretical frameworks.

3. Provide Context about the Network: This manuscript did not provide the reader with any insights to suggest that the network was successful in achieving the goal – lower case fatality rates. The reader should be informed if this manuscript (evaluation of a network) is reviewing a failed or successful network.

**Response:** This is a very important observation, thanks. We evaluated a network whose goal was lower case fatality rates; and, the other 8 manuscripts in this collection all cover different aspects of the network – including network effectiveness, shortcomings and explanatory factors across the different contexts or case study countries. However, we very much appreciate the reviewer’s observation and guidance – and have now provided additional signposting to these complementary manuscripts on Page 4, Lines 91 – 93 (see also S1_Text supplementary material).

4. Strengthen the Methods: Based on the title, this manuscript seeks to provide a “how to” yet the methods section is sparse. In particular, the analytical methods are largely missing in the methods section. The authors explain that in addition to individual country analyses, a series of core questions were asked. The authors explain the team composition and how the team communicated yet it is not clear how the authors understood success or failure of the network.

**Response:** In trying to provide insights and lessons learned from our evaluation experience, we explain the processes and structures which guided our work in narrative manner which harvests learning and documents it for reference. As a core part of the “how to”, this paper provides more operational than analytical insights; the specifics of whether the network was successful or not are directly tackled in the Collection’s other eight (8) manuscripts and are not part of this manuscript’s central focus. Also, to mention here that over the course of the manuscript’s numerous (more than 5) revisions following reviews, the methods section has continued to evolve and, as authors, we feel that we have reached that point which communicates what we intended to share with the scientific community (kindly please review all of the earlier revisions of our paper). We have also tried to make clear what we perceive to be our limitations in the conduct of this evaluation.

5. Check Title Clarity: The title could be perceived as broader than the manuscript's content. It may benefit from a revision to directly reflect the paper's essence, which is a reflective analysis of the evaluation process of the QCN.

**Response:** Thank you for this comment. We have now amended the title from *“How to evaluate a multi-country implementation-focused network: Lessons from the Quality of Care Network (QCN) evaluation”* to *“How to evaluate a multi-country implementation-focused network: Reflections from the Quality of Care Network (QCN) Evaluation”*

6. Revisit Innovation Claims: I am skeptical that there was innovation in the ways of working. During the time of this evaluation, the world was undergoing a massive shift towards video/teleconferencing linked to COVID. Against this backdrop, the authors are not clear how this work is innovative.

**Response:** At the time of the actual evaluation, when little was known about how to go about COVID related limitations in the conduct of multi-context research, our approach felt innovative because we were trying out some new things and approaches which later on came to be widely adopted and therefore common, even normal as we know them now. So, in retrospect and at this point in time, we agree that it is fair to revisit these innovation claims. We have now amended and used the word “adaptation” instead of “innovation” – which we hope addresses this comment. The changes can be found across the entire manuscript from Page 2 (Line 47), Page 2 (Line 58), Page 7 (Lines 163,176) and Page 12 (Line 275).

7. Provide Evidence: The section titled “what went well” includes statements about the network (e.g. co-production) with external citations. However, this evaluation is largely a qualitative analysis, yet the authors do not provide qualitative evidence to back up these statements about what went well.

**Response:** Thank you for this comment. Our explanation for why the section titled “what went well” does not provide qualitative evidence is two-fold: 1) It falls under what would be the equivalent of a ‘normal’ paper’s “discussion” section. We are not sure if the current reviewer was provided background information about the history of this particular manuscript including the fact that PLOS GPH does not publish ‘methods’ papers – and, in particular, how we struggled to fit this paper very well within the structure of a typical research manuscript, as well as how multiple reviewers kept suggesting changes its structure and content. That partly explains this dilemma; 2) Secondly, this is a purely reflective piece from/by the evaluation team – therefore almost everything we write about is from and about us as a team, i.e. from us, the authors of this manuscript. We believe this, in itself, is qualitative evidence.

8. Consider Additional Literature: The authors may strengthen this manuscript by reviewing literature from similar networks that do not seem to be featured here (Joint Learning Network, Salud Mesoamerica network). In addition, there is a robust literature on network theory outside of healthcare which, if leveraged, could ground this analysis more holistically.

**Response:** Thank you for this comment. We have addressed this in our responses to comments 1 and 2 above, i.e. comparison to other networks is beyond the scope of this paper, and we cover network theory in another of our papers in our collection of 9 papers.
